# Progress in Therapeutics

**Published:** 1893-07

**Authors:** 


### Editorial, Etc.

PROGRESS IN THERAPEUTICS.—It is really astonishing how little injury to the constitution is done by what all must admit to be over-drugging. The number and variety of drugs is a confession of insufficiency. Dr. H. C. Wood, in his standard work on "Therapeutics: its Principles and Practice," asks: "What has clinical therapeutics established permanently and indisputably?" and answers, "Scarcely anything beyond the primary facts that quinia will arrest an intermittent, that salts will purge, and that opium will quiet pain and lull to sleep." . . "Yet with what a Babel of discordant voices does it [the profession] celebrate its two thousand years of experience."

Is there any solid basis for medical practice? Is therapeutics grounded on science? Must it be frankly admitted that the members of the medical profession are only empirics, who having tried this, or that drug, or at all events their predecessors having tried it, have found it to succeed? Must we wait until we know what life is, before we can expect to know how drugs prolong it?

Many years ago a physician and chemist proposed to prove that it is neither in accordance with the rules of allopathy or of homœopathy, but in "endowing and enriching" the body that cures are performed. The art of healing is passing through a crisis. Medicine is now entering upon a biological phase, in which disease is regarded as an imperfect form of undeveloped vitality, as a loss of something present in health. Under the light of advancing physiology the end and aim of happy treatment is essentially an addition; an endeavor to retain, to restore, to develop into fuller life those identical morbid substances and processes which have hitherto been uniformly condemned to expulsion—'Tis life, more life, for which we pant." Now you read of medicines advised to support the strength of the patient, of restoratives which promote constructive metamorphosis, termed tonics in the older classifications; whereas formerly the most active debilitants were named with the avowed purpose of weakening the disease. Now the articles of materia medica brought into common use are cod-liver oil, hypophosphite of lime, phosphates of iron, pepsin, etc., whose intention is to form a basis of new cell growth. There is another interesting class of substances embracing the iodides and bromides, which are applicable to various ailments, although the connection is not obvious, such as syphilis, epilepsy, neuralgia, gout, hysteria, lead poisoning, etc.

The surgeon, too, when the skin is lost or wounded builds up as good a restoration or imitation as he can. A bit of clean skin, stripped from the patient, or a friend, and planted among torpid granulations, sticks, unites, feels and becomes with its new home one flesh not to be put asunder. Truly, not only disease but health is contagious. Better still it is infectious.

That which above all other things has contributed to change of practice is the gradual change in ideas respecting inflammation, together with the distinction made between disease and its cause, arising out of the attention paid to morbid germs; and the adoption of the motto, "Prevention is better than cure." R. G.

